# Licocalchone-C Extracted from *Glycyrrhiza Glabra* Inhibits Lipopolysaccharide-Interferon-γ Inflammation by Improving Antioxidant Conditions and Regulating Inducible Nitric Oxide Synthase Expression

**DOI:** 10.3390/molecules16075720

**Published:** 2011-07-06

**Authors:** Sara Franceschelli, Mirko Pesce, Isabella Vinciguerra, Alessio Ferrone, Graziano Riccioni, Antonia Patruno, Alfredo Grilli, Mario Felaco, Lorenza Speranza

**Affiliations:** 1 Department of Human Movement Sciences, University G. D’Annunzio of Chieti, 66123, Italy; E-Mails: s.franceschelli@unich.it (S.F.); mirkopesce@unich.it (M.P.); i.vinciguerra@unich.it (I.V.); a.ferrone@hotmail.it (A.F.); algrilli@unich.it (A.G.); 66123.mfelaco@unich.it (M.F.); 2 Cardiology Unit, San Camillo de Lellis Hospital, Manfredonia, Foggia, Italy; E-Mail: g.riccione@yahoo.it (G.R.); 3 Department of Drug Sciences University G. D’Annunzio of Chieti, 66123, Italy; E-Mail: antoniapatruno@unich.it (P.A.)

**Keywords:** licochalcone, inducible nitric oxide sinthase, antioxidant properties

## Abstract

The genus *Glycyrrhiza* consists of about 30 species, amoung these, *G. glabra* is the source of several phenolic compounds, known as flavonoids, such as licoagrodin, licoagrochalcones, licoagroaurone and licochalcone C, kanzonol Y, glyinflanin B and glycyrdione A, which have shown various pharmacological activities, including antitumor, antiparasitic, antileishmanial, anti-ulcer and antioxidative effects. Among these compounds, licochalcone C was isolated but its biology has not been fully examined. In our study we reproduced an inflammatory state by treating THP-1 (human myelomonocytic leukaemia) cells with pro-inflammatory stimuli, such as LPS and IFN-γ and we investigated the possible antioxidant activity of licochalcone C at a concentration of 50 μM. Our results show that treatment with licochalcone C attenuates the LPS-IFN-γ-induced inflammatory response by significantly decreasing the expression and activity of iNOS via NFκB (nuclear factor kappa-B), by influencing extracellular O_2_^−^ production, and by modulating the antioxidant network activity of SOD (superoxide dismutase), CAT (catalase) and GPx (glutathione peroxidase) activity. Based on these results we hypothesize that Licochalcone C has antioxidant properties since it reduces the production of superoxide radicals and consequently reduces the activity of iNOS.

## 1. Introduction

Licorice (*Leguminosae*) is a perennial herb that grows in Southern Europe, central Russia, Turkey, Iraq and Iran. It has been used in traditional medicine for the roots of several *Glycyrrhiza* species since ancient times. The genus *Glycyrrhiza* consists of about 30 species, among these, *G. glabra* is the source for several phenolic compounds, known as flavonoids, such as licoagrodin, licoagrochalcones, licoagroaurone and licochalcone C, kanzonol Y, glyinflanin B and glycyrdione A, which shown various pharmacological activities, including antimutagenic, antiparasitic, antileishmanial, anti-ulcer and antioxidative effects. [1,2,3,4,5,6].

Among these compounds, the flavonoid licochalcone C has been isolated, but its biology has not been fully elucidated [7]. Over 4,000 flavonoids have been identified in plants [8]. The commonly used herbs that provide substantial amounts of flavonoids include chamomile, dandelion, ginkgo, green tea, hawthorn, licorice, passionflower, milk thistle, onions, rosemary, sage, thyme, and yarrow. Flavonoids have extensive biological properties that promote human health and help reduce the risk of disease [9,10]. Remarkably, these compounds have been reported elsewhere to inhibit nuclear factor-kappa B (NF-κB) activation, which is considered as an important underlying mechanism of the anti-inflammatory activities of flavonoids. [11,12].

The NF-κB family of transcription factors play the most important role in the immune system. NF-kB is known to regulate the expression of many genes involved in immune and inflammatory responses [13]. Importantly, the NF-κB binding site has been identified on the inducible nitric oxide synthase (iNOS) promoter and plays a role in the induction of this enzyme. 

iNOS, the enzyme responsible for the generation of high amounts of nitric oxide (NO) from the aminoacid L-arginine, is expressed in several cell types after its transcriptional activation [14]. Importantly, aberrant expression of iNOS contributes to the pathogenesis of many human diseases, such as septic shock and rheumatoid arthritis. Additionally, the induction of iNOS by different stimuli leads to organ destruction in some inflammatory and autoimmune diseases [15]. During the inflammatory process, high amounts of NO and reactive oxygen species (ROS), as superoxide anion (O_2_^−^), hydrogen peroxide (H_2_O_2_) and hydroxyl radical (OH^−^), contributing to intracellular destructive mechanisms. In addition, NO produced by the enzymatic catalysis of iNOS, presents a high affinity for superoxide which contributes to alter the cellular redox state through the formation of peroxynitrite (ONOO^−^) [16,17]. An increasing number of therapeutic agents have been reported to inhibit the expression of iNOS and exert their anti-inflammatory effect by inhibiting iNOS. Furthermore, natural compounds are increasingly used in inflammatory disorders because of their fewer side effects and lower cytotoxicity [18,19]. In this study, we isolated a natural compound, licochalcone C, from *G. glabra* and we conducted a series of experiments using human acute monocytic leukemia cell line (THP-1) that mimic the cellular inflammation model to explore potential mechanisms for the activity of the phenolic compounds. We investigated the putative antioxidant effects of licochalcone C evaluating both the enzymatic activity of the antioxidant enzymes (superoxide dismutase, SOD; catalase, CAT; glutathione peroxidise, GPx) that iNOS modulation via activation of NFkB transcription.

## 2. Results and Discussion

### 2.1. Cytotoxicities of Licochalcone C

**Figure 1:**
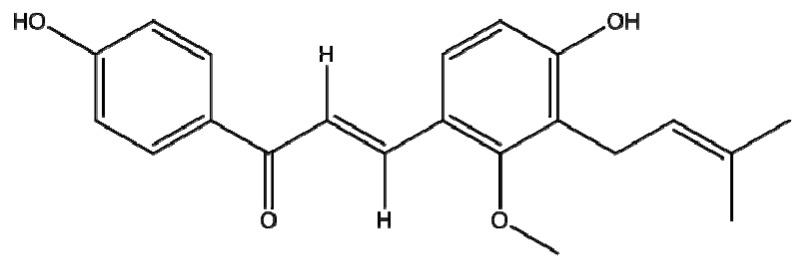
Licochalcone C structure.

In this study, we isolated licochalcone C (Figure 1) and examined its effects on LPS (10 µg/mL) + IFN-γ (20 ng/mL)-induced inflammation in a THP-1 cell line model. 

First, we evaluated the cytotoxicity of licochalcone C in THP-1 cells by an MTT assay before and after 24 hr of incubation, respectively, and it was observed that this phenolic compounds did not affect cell viability between the before and after 24 h of incubation (Table 1). For all further experiments, only a non toxic concentration of 50 µM licochalcone C were used.

### 2.2. Antioxidant Activity of Licochalcone C

Many phenolic compounds present in medicinal plants possess antioxidant and anti-inflammatory activities and previous reports have evidenced that the constituents of *Glycirrhyza* were effective in preventing microsomal lipid peroxidation induced by Fe (III)-ADP/NADPH and licochalcone B and D exhibited potent antioxidative and superoxide scavenging activities [20].

**Figure 2:**
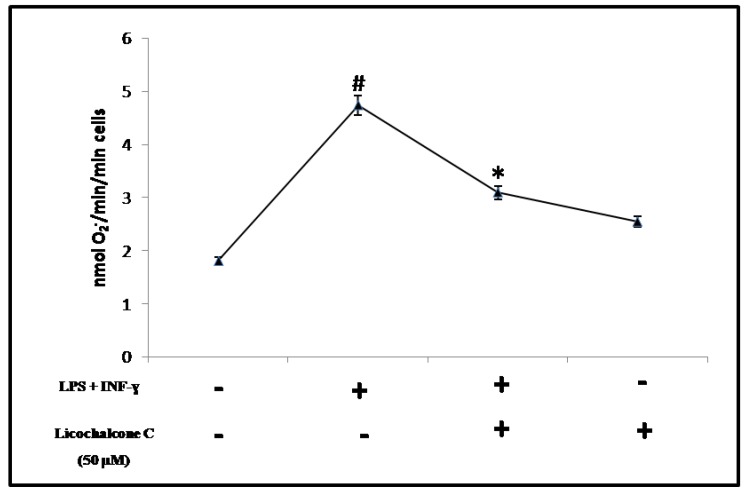
Determination of O_2_^−^. THP-1 cells treated with licochalcone C 50 µM reduced the extracellular production of superoxide anion (O_2_^−^), respect to cells incubated with pro-inflammatory cytokines (LPS - 10 µg/mL and INF-γ - 20 ng/mL). All the data of the comparative studies had a p value (* ANOVA < 0.005 *vs.* THP-1 cells incubated with LPS+INF-γ; ^#^ ANOVA < 0.05 *vs.* THP-1 control cells).

ROS are a major constituent of inflammation that can affect normal cellular function and have pathogenic consequences. The burst of activated, oxygen-derived free-radical species is responsible for peroxidation of cell membranes, resulting in tissue edema, and protein and enzyme degradation. They can also compromise cellular repair mechanisms, cause premature aging, and trigger apoptotic processes [21]. Quenching ROS production can decrease inflammation and subsequent tissue damage. The first line of cellular antioxidant defence consist of free radical scavenging enzymes, such as SOD, CAT and GPx. Thus, to eliminate free radicals, these cellular antioxidants play an important role in maintaining a redox equilibrium under normal physiological conditions but act in first line defence against excessive production of free radicals [22]. SOD catalyzes the dismutation of O_2_^−^ to H_2_O_2_. In turn, l’H_2_O_2_ is reduced to O_2_ and H_2_O by the enzyme CAT and GPx [23]. Initially, extracellular superoxide radical levels were determined spectrophotometrically (Figure 2).

**Figure 3:**
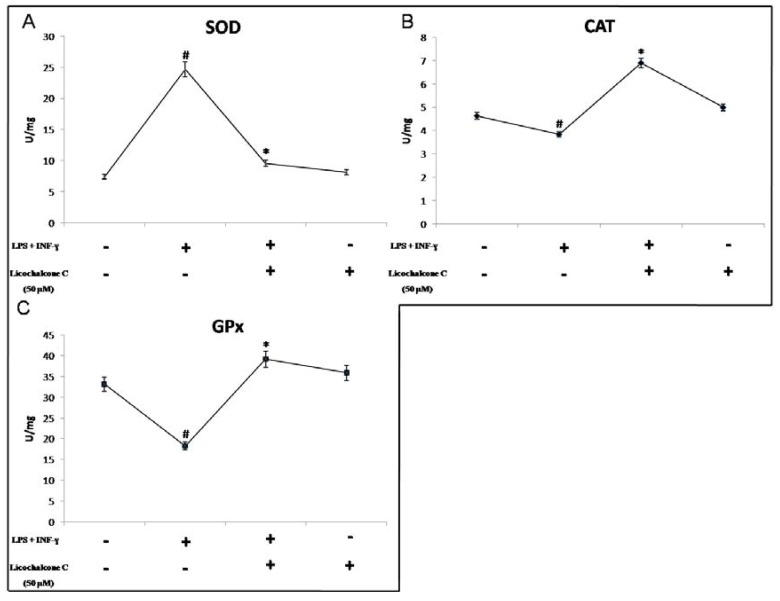
Evaluation of SOD, CAT and GPx activity. Activity of SOD (A), CAT (B) and GPx (C) is illustrated; Licochalcone significantly influence SOD, CAT, and GPx activities levels. Data are means +/− SD from four individual experiments (* p < 0.05 for cells co-stimulated *vs.* LPS+ INF-γ. treated cells).

In THP-1 cells treated with proinflammatory cytokines there is a marked production of such radicals, highlighting the fact that under similar conditions elevated levels of oxidative stress demonstrated by cells coincubated with cytokines, the dismutase reaction with SOD is extremely elevated (Figure 3A). 

This increase in SOD activity involves an accumulation of H_2_O_2,_ not adequately metabolized from CAT and GPx, with consequent intracellular accumulation of toxic radicals that, as also shown in other studies, may lead to the activation of inducible enzymes, whose catalytic product can reversibly inhibit antioxidant enzymes such as CAT and GPx (Figures 3B, 3C) [24.^25^]. Treatment with licochalcone C shows a significant reduction in the production of O_2_^−^ and as shown in Figure 2, appears to be comparable to baseline values. Taken together, these results strongly suggest that licochacone C has the ability to decrease oxidative stress produced in vitro on THP-1 cells through the activation with proinflammatory cytokines, elucidating the antioxidant profile of this natural compound.

### 2.3. Influence of licochalcone C on iNOS signaling via NFkB

**Figure 4:**
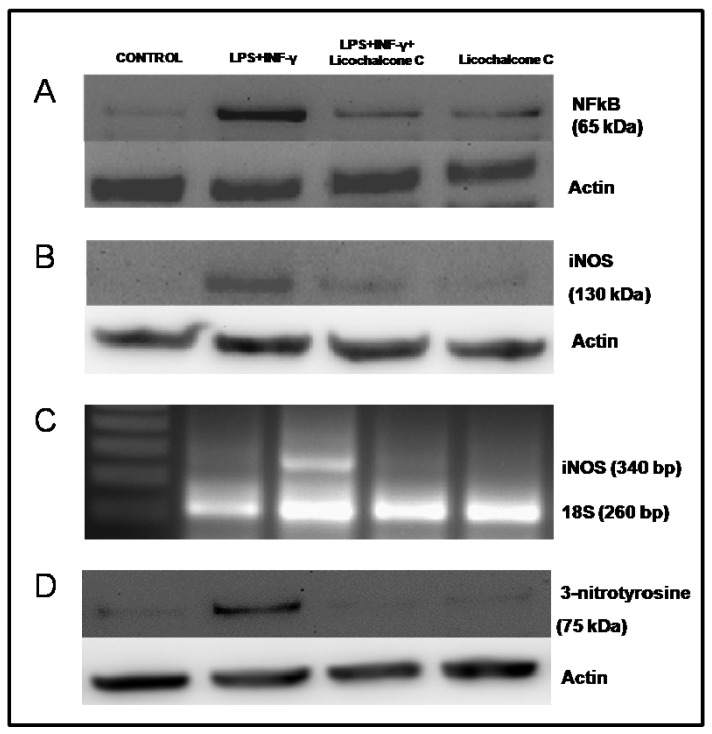
Influence of licochalcone C on iNOS signaling via NFkB. **A**. Western blot detection NFkB nuclear protein extracts from THP-1 cells treated with LPS (10 µg/mL)+ INF-γ (20 ng/mL) and licochalcone C (50 µM), either individually or simultaneously (both). **B**. Western blot analysis of LPS+ INF-γ- treated THP-1 cells, with and without licochalcone C (50 µM) were analyzed for total iNOS protein. Licochalcone C treatment reduced iNOS protein expression respect to cells LPS+ INF-γ-stimulated. **C**. Effects of licochalcone C, either alone or in combination, on the up-regulation of iNOS mRNA expression (examined by RT-PCR) induced by LPS and INF-γ in THP-1 cells. Gel electrophoresis samples of semi-quantitative RT-PCR for determination of iNOS mRNA levels. 18 S was internal standard. The comparison of the relative density of iNOS/18S PCR products (control, LPS + INF-γ, LPS + INF-γ + licochalcone C and licochalcone C alone) evidencing that licochalcone C treatment induced a down-regulation of iNOS mRNA expression. **D**. Western blot analysis of 3-nitrotyrosine shows the effects of licochalcone C on 3-nitrotyrosine protein content in THP-1cell lisate. Nitrotyrosine was detected in cytokine-treated THP-1 cells but not in untreated cells and in cells treated with licochalcone C.

Several non-enzymatic (e.g., glutathione, flavonoids, and vitamins A, C, and E) as well as enzymatic scavengers of ROS (e.g., SOD, CAT, and GPx) prevent the accumulation of ROS. Unfortunately, these defense mechanisms are not always adequate to counteract the toxic effects of ROS, resulting in what is termed a state of oxidative stress. Phenolic compound via their antioxidant effects in protecting cellular components against ROS [26]. Antagonizing ROS production by several antioxidants abrogated the activation of NF-kB [23]. Cells that have an excessive production of free radicals lead to an activation of a series of transcription factors (e.g., AP-1, NFkB, Nrf2) associated with induction of genes coding for proteins, such as cycloxygenase (COX-2) and iNOS-2, closely involved in inflammatory responses. In our experimental model, it is possible to observe that in activated THP-1, an altered redox state leads to a high expression of the transcription factor NFkB (Figure 4A) directly involved in the modulation of the inducible isoform of NOS [27,28].

**Figure 5:**
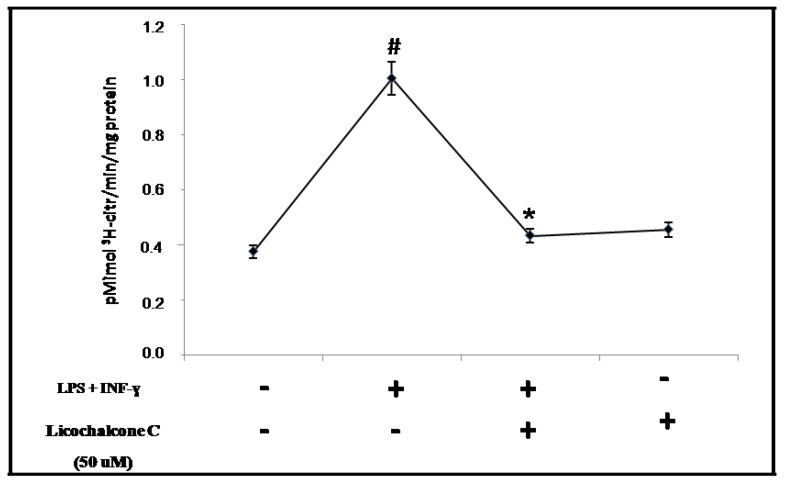
Citrulline synthesis. iNOS activity was analyzed in control, stimulated and co-stimulated cells with or without LPS+INF-γ after licochalcone C treatment by measuring [3H]-arginine to [3H]-citrulline conversion. NOS activity are reduced in THP-1 cell line co-treated with citokynes and licochalcone C. Error bars represents standard deviation from the mean (n = 4, * p < 0.05 *vs.* THP-1 stimulated with LPS+INF-γ; ^#^ p < 0.05 *vs.* THP-1 control cells).

In fact, as in the RT-PCR and Western blot analysis of activated THP-1 cells, there is an up-regulation of both the transcript as well as the iNOS protein and thus increased activity of this enzyme responsible for the production of large quantities of NO (Figures 4B, 4C; Figure 5) that has a high affinity for the O_2_^−^ radical, producing stronger oxidant ONOO^−^ (Figure 4D), as also demonstrated by evaluating the expression of a specific marker of the main production of peroxynitrite [29,30].

Drugs that inhibit iNOS expression and/or enzyme activity, resulting in decreased NO generation, may be beneficial in treating diseases caused by an overproduction of NO [31]. The densitometric analysis showed a diminished expression and activity of iNOS in the co-stimulated cells with cytokines and licochalcone C at a concentration of 50 µM, respect to cytokines alone (Table 2).

Treatment with licochalcone C has strong antioxidant properties, since it reduces the production of ROS and restores physiological enzymatic activity in the network, it down-regulates the transcription factor NF-kB, thereby decreasing the production of a highly reactive species, such as that ONOO^−^. Because of its high reactivity it generates extensive lesions in the same cells that produce it.

## 3. Experimental

### 3.1. General

Licochalcone C was extracted as described by Yoon *et al*. [32]. Dried roots (2 kg) from *Glycyrrhiza glabra* were grinded into fine powder in liquid nitrogen, with a mortar and pestle. Afterwards, two consecutive extraction with 4 L of boiling distilled water for 2.5 h were done. Fatty acids were removed from the combined water extract with 2 L of *n*-hexane. The defatted solution was extracted three times with 2 L of dichloromethane and then, the combined organic extract was evaporated. The residue was dissolved in chloroform and loaded onto a silica column. The elution solvent system used was a mix of n-hexane-ethyl acetate-methanol 2:1:0.1. Fractions were analyzed on TLC and those presenting large spots near the *R_f_* of pure licochalcone A (Sigma) were combined, concentrated and loaded onto a second silica column and eluted with chloroform-methanol (10:1) eluent system to obtain 15 mg of licochalcone C. Purity was checked with a HPLC system (P/U-2080, Jasco) equipped with a diode array detector (MD-2010, Jasco) using a Tracer Hypersil ODS (5 µm) C_18_ column (10 × 250 mm). The compound was obtained with a purity of 98% and its structural identity was confirmed by H^1^-NMR (Varian 300 MHz spectrometer); H-NMR (CDCl_3_), δ 1,70 (3H, s, H-4"), δ 1,82 (3H, s, H-5"), 3,21 (2H, d, H-1"), 3.83 (3H, s, OMe), 5,75 (1H, t, H-2"), 6.76 (1H, s, H-5), 6.91 (2H, d, *J* = 8.7 Hz, H-3′, 5′), 7.60 (1H, d, *J* = 15.5 Hz, H-α), 7.80 (1H, s, H-6), 7.96 (2H, d, *J* = 8.7 Hz, H-2′, 6′), 8.00 (1H, d, *J* = 15.5 Hz, H-β).

### 3.2. Cell culture

Cell culture THP-1 (human myelomonocytic leukaemia) cell line (ATCC number TIB-202), were cultured at a density of 10^6^ cells/mL in RPMI 1640 medium supplemented with 10% heat-inactivated FCS, 100 ng/mL streptomycin, 100 U/mL penicillin, and 2 mM L-glutamine in a 5% CO_2_ air humidified atmosphere at 37 °C and passaged every 4 to 5 days. The cell viability, determined by trypan blue exclusion was >99%. Cells were seeded onto six-well tissue culture plate and incubated overnight at 37 °C in a humidified atmosphere of 5% CO_2_. Cell viability was not influenced in field exposed non-stimulated cells nor LPS-stimulated cells. More than 98% of cells were viable, as determined by trypan blue dye exclusion at the beginning of the culture, and more than 90% were viable before cells were collected. Cell were divided in: (1) control cells; (2) cells stimulated with LPS (10 µg/mL) + IFN-gamma (20 ng/mL); (3) cells stimulated with LPS (10 µg/mL) IFN-gamma (20 ng/mL) and licochalcone C (50 µM); (4) cells stimulated only with licochalcone C (50 µM) for 24 hours. The dose-effect relationship of the licochalcone C was previously determined in a set preliminary of experiments, and it was found that 50 µM was associated with the down expression of iNOS and no cellular toxicity. Cell viability was determined by trypan blue day exclusion and MTT assay (Biotium, Hayward, CA, USA).

### 3.3. Determination of O_2_^−^

Production of O_2_^−^ was determined spectrophotometrically (Hewlett Packard 8452 A) by monitoring the reduction of ferricytochrome c (Type VI, Sigma) at 550 nm, as described by Pritchard [33] Ferricytochrome c (final concentration, 50 μmol/L) was added directly to the cuvette containing the cells and Dulbecco’s phosphate buffered saline (DPBS) (final volume 1 mL), in the presence or absence of superoxide dismutase [(SOD), 350 U/mL], and changes in absorbance were followed for 10 minutes. Rates of O_2_^−^ production were calculated on the basis of the molar extinction coefficient of reduced ferricytochrome c [ε = 21,000 cm ^−1^ (mol/L) *^−^*^1^]. Cell counts were used to calculate results as nanomoles O_2_^−^ per 10^6^ cells per minute.

### 3.4. Cu, Zn-Superoxide Dismutase Activity

SOD activity was determined as described by Sun and Zigman [34]. The assay mixture contained 50 mM sodium carbonate buffer, pH 10, epinephrine 0.1 mM (Sigma), and tissue fraction (containing about 1-50 μg of protein) in a final volume of 2.5 mL. The inhibitory effect of SOD on the autoxidation of epinephrine, with the use of 1.25 mM KCN to discriminate the CN- insensitive MnSOD from the CN- sensitive Cu, ZnSOD was assayed spectrophotometrically at 480 nm at 25 °C. Percentage inhibition values were converted into activities by using a purified Cu, Zn bovine SOD as standard (Sigma). One unit of SOD is the amount of enzyme required to halve the rate of substrate auto-oxidation.

### 3.5. Catalase Activity

CAT activity was measured spectrophotometrically [35]. The decomposition of H_2_O_2_ was monitored continuously at 240 nm. The assay mixture in a final volume of 3 mL contained 10 mM potassium phosphate buffer, 10 mM H_2_O_2_ and 1.5-11 μg of protein of enzymatic extract. CAT units were defined as 1 μmole H_2_O_2_ decomposed/min at 25 °C.

### 3.6. Glutathione Peroxidase Activity

Quantification of GPx activity was evaluated using the Paglia and Valentine method as modified by Di Ilio *et al*. [36,37]. The activity of the Se-dependent GSH peroxidase was measured with H_2_O_2_ (0.25 mM) as substrate. The oxidation of NADPH was followed at 25 °C on a Hewlett Packard spectro-photometer at 340 nm. One unit was defined as 1 μmol of GSH oxidized min.

### 3.7. Protein Extraction and Isolation of Nuclei

THP-1 cells were washed once in cold phosphate-buffered saline (PBS; 0.5 mol L^−1^ sodium phosphate, pH 7.5) and harvested by gentle scraping, and used to prepare total protein or nuclear extracts. Total protein extracts were prepared by treating cells with lysis buffer [50 mmol L^−1^ Tris–HCl pH 7.5, 0.4% Nonidet P-40 (NP-40), 120 mmol L^−1^ NaCl, 1.5 mmol L^−1^ MgCl_2_, 2 mmol L^−1^ phenyl-methylsulphonyl fluoride (PMSF), l μg mL^−1^ leupeptin, 3 mmol L^−1^ NaF and 1 mmol L^−1^ dithiothreitol] for 30 min at 4 °C. For preparation of nuclear extract the cells, resuspended in 10 mM Tris–HCl, pH 7.4, 10 mM NaCl, 2 mM MgCl_2_, 0.6% Triton X-100, 1.0 mM PMSF, 1 mg/mL leupeptin, and aprotinin, were incubated at room temperature for 2 min, then cooled on ice for 5 min. After five passages through a 22-G needle, MgCl_2_ concentration was adjusted to 5 mM. Nuclei were obtained by centrifuging the suspension at 1200*g* for 15 min and cytoplasmic fractions consisted of the postnuclear supernatants. Nuclei were then harvested in RIPA buffer (1×PBS, 1% Nonidet P-40, 0.5% sodium deoxycholate, 0.1% SDS, 10 mg/mL PMSF, 100 mM sodium orthovanadate, and 1 mg/mL leupeptin and aprotinin).

### 3.8. Western blot Analysis for iNOS, NF-κB and 3-nitrotyrosine

Determination of iNOS, 3-nitrotyrosine and NFkb proteins were performed in two series of protein extracts from THP-1 cell line by Western blotting. Fifty μg cytoplasmatic and 10 μg nuclear proteins, quantified by spectrophotometric assay (HP 8452A, Palo Alto, CA, USA) using the Breadford method, were separated by electrophoresis in a 7.5% sodium dodecyl sulfate–polyacrylamide gel (SDS–PAGE; Bio-Rad, Hercules, CA, USA) and transferred at 4 °C to nitrocellulose membrane (Bio-Rad, Hercules, CA, USA) in glycine–methanol buffer. Nitrocellulose was then blocked in Tris-buffered saline (TBS)–milk and incubated, overnight, with various primary antibodies: anti-human iNOS (Santa Cruz Biotechnology, Santa Cruz, CA, USA), monoclonal anti-3-nitrotyrosine (Santa Cruz Biotechnology, Santa Cruz, CA, USA) and anti-human NF-κB (Santa Cruz Biotechnology). The nitrocelluloses were then washed in TBS, incubated with a secondary antibody conjugated with alkaline phosphatase for 2 h, washed again, and developed in an alkaline buffer with nitroblue tetrazolium (NBT) as substrate (Alkaline Phosphatase Conjugate Substrate Kit, Bio-Rad, Hercules, CA, USA). Antibody anti-β-Actin (Sigma), and Actin (Sigma), were used as an internal standard. The densitometric analysis of Western blots was performed using a computerized densitometric system (Bio-Rad Gel Doc 1000, Milan, Italy).

### 3.9. Semi-Quantitative Reverse Transcription-Polymerase Chain Reaction for iNOS

Semi-quantitative reverse-transcribed polymerase chain reaction (RT-PCR) was used to determine mRNA levels of the inducible Nitric Oxide Synthase. Total RNA was extracted using 1 mL ULTRASPEC-RNA (Biotecx, Lab., Inc. Huston, TX, USA), as recommended by the manufacturer. RNA was dissolved in diethyl pyrocarbonate (DEPC)-treated water and quantified spectrophotometrically at 260 nm.

First-strand cDNA was generated by adding RNA (1 μg) to a mixture containing 1 mM deoxy-nucleoside-triphosphates (d-NTP), 1U/μL RNase inhibitor, 2.5 U/μL moloney murine leukemia virus reverse transcriptase, 2.5 μM oligo-dt, 5 mM MgCl_2_, 10x PCR buffer in a final volume of 20 μL. Reverse transcription was performed at 42 °C for 1 hr followed by heat inactivation of reverse transcriptase at 92 °C for 10 min. 18S was amplified from the same amount of RNA to correct for variation of different samples. PCR amplification was performed using a Programmable Thermal Controller (MJ Research, Inc. Waltham, MA, USA). The PCR solution contained 10 μL of first-strand cDNA, 4 μL 10x PCR buffer, 2 mM MgCl_2_. The following primer pairs were used: sense 5’-CGT AAA GAC CTC TAT GCC AA-3’ and antisense 5’-AGC CAT GCC AAA TGT CTC AT-3’ for iNOS, and 18S primers, sense 5’-TAC GGA GCA GCA AAT CCA C-3’ and antisense 5’-GAT CAA AGG ACT GCA GCC TG-3’, 2U Termophylus Acquaticus (Taq) DNA polymerase (Celbio, Milan, Italy), and water to a final volume of 50 μL. These samples were overlaid with mineral oil and subjected to 35 cycles at 95 °C for 60 sec, 60 °C for 60 sec, and to one cycle at 72 °C for 7 min for iNOS and 40 cycles at 95 °C for 60 sec, 58 °C for 60 sec, and to one cycle at 72 °C for 7 min for 18S. PCR products were run on 2% agarose gel electrophoresis and photographed after ethidium bromide staining under UV light. Bands on the gel were scanned using a computerized densitometric system (Bio-Rad Gel Doc 1000, Milan, Italy).

### 3.10. Citrulline Synthesis 

The measure of the conversion of L-arginine to L-citrulline is a standard assay method currently used to quantitative NOS activity. Briefly, 10 μL of radioactive arginine, L-(2,3,4,5–^3^H) arginine monohydrochloride 64 Ci/mM, 1 μCi/μL (Amersham, Arlington Heigths, IL, USA) and 50 μL NADPH 10 mM were added to each cell homogenate samples and incubated for 30 min at room temperature. After incubation, the reactions were stopped with 400 μL of stop-buffer (50 mM HEPES, pH 5.5, 5 mM EDTA) and added of the equilibrated resin into each sample. The equilibrated resin bound unreacted arginine. After centrifugation, the radioactivity corresponding to L-(^3^H)-citrulline was measured with liquid scintillation spectrometry. Calcium was omitted from these incubations to favour the determination of the calcium independent iNOS isoform.

### 3.11. Statistical Analysis

The results were expressed as mean ± SD. Statistical analysis was performed using the analysis of variance (ANOVA). Probability of null hypothesis of <5% (p < 0.05) was considered statistically significant.

## 4. Conclusions

As many studies have demonstrated that the use of several compounds of plant origin show a multiplicity of pharmacological activities against a number of pathological conditions, in this study we isolated the structural isomer of licocalchone A, licocalchone C, from licorice, which in the literature was shown to exhibit various biological activities [38,39,40,41]. Traditionally, these compounds have been used in northeast Asia for the treatment of gastric and duodenal ulcers, bronchial asthma, inflammation, and other diseases. Much of the recent research on the constituents of licorice has resulted in finding the pharmacological importance of various phenolic compounds [42,43,44]. Peripheral blood monocytes may reflect the bias of the donating individual, and it is hard to assess the role of individual effects on the results of tests. PBMCs are almost impossible to standardize for use in standard tests to evaluate biological response. Because of the shortcomings of PBMCs, the THP-1 monocyte cell line has been used extensively as a model for testing the drug. The THP-1 was more sensitive than PBMCs in cytotoxic evaluations, but provided an identical ranking of relative cytotoxicities with significantly less variation. In this model, an inflammatory state has been reproduced in THP-1 with the stimulation of LPS and IFN-γ that determine activation of the iNOS gene via NF-kB, resulting in a high production of nitric oxide [23]. Our hypothesis is that treatment with the licocalchone C, resulting in a reduction of the superoxide radical, restores a physiological condition that down regulates iNOS. At the cellular level this lead to a significant reduction of NO, which influences: (1) production of peroxynitrite, as we have demonstrated in the treatment there is nitrosylation of proteins; (2) restoration of the activity of CAT and GPx. In fact, several experimental evidences suggest that in a state of oxidative stress (as in our model) there may be an interaction between NO of the heme catalase, resulting in a reversible inhibition of the enzyme [25]. Furthermore, as evidenced by Dobash *et al*. [24] we found a temporary inhibition of GPx probably through the interaction of NO with the thiol group of the enzyme. Therefore, our results show that the excessive NO production in vitro by endogenous iNOS in response to cytokines leads to alterations of the redox state through the down-regulation of catalase, GPX and the up-regulation of CuZn-SOD. This alteration of cellular redox may play a pivotal role in the pathophysiology associated with the induction of iNOS. Our results show for the first time that treatment with licocalchone C could attenuate LPS-IFN-γ-induced inflammatory response via the modulation of oxidative stress through decreasing O_2_^−^ production and modulation of antioxidant network activity of SOD, CAT and GPx in a biological model. Moreover, licocalchone C, by scavenging ROS, is responsible for the inhibition of iNOS via NFkB in THP-1 cell line. Decreasing NO production, significantly prevents the formation of ONOO^−^. Since both the NO and NF-kB are important inflammatory mediators, they play key roles in modulating the formation of various radical species during the oxidative action of licocalchone C, and may represent a new target of action against inflammatory diseases which have in common an alteration of the redox status.

## Figures and Tables

**Table 1:** Licochalcone C concentration-effect curve on cell viability.

	Before incubation	After 24 hr of incubation	
Control cells	96.2%	95%	
Cells + 500 μM Licochalcone C		91%	p < 0.05
Cells + 100 μM Licochalcone C		91.4%	p < 0.05
Cells + 50 μM Licochalcone C		94.4%	p < 0.05

**Table 2:** Densitometric analysis. The quantitative densitometry of NFkB, iNOS and 3-nitrotyrosine of samples collected from controls, LPS + INF-γ, LPS + INF-γ + Licochalcone C and Licochalcone C alone. Data are expressed as mean ± SD (* p < 0.05 *vs.* cells incubated with pro-inflammatory cytokines).

	CONTROL	LPS + INF-γ	LPS + INF-γ + Licochalcone C	Licochalcone C
**NFkB** Western Blotting	0.23 ± 0.04	1.17 ± 0.04	0.39 ± 0.03*	0.31 ± 0.03
**iNOS** Western Blotting	0.17 ± 0.05	1.01 ± 0.03	0.28 ± 0.04*	0.23 ± 0.03
**iNOS** rt-PCR	0.25 ± 0.04	0.62 ± 0.04	0.15 ± 0.02*	0.16 ± 0.03
**3-Nitrotyrosine** Western Blotting	0.23 ± 0.06	1.12 ± 0.02	0.27 ± 0.05*	0.17 ± 0.04
